# Elucidating negative symptoms in the daily life of individuals in the early stages of psychosis

**DOI:** 10.1017/S0033291720001154

**Published:** 2021-11

**Authors:** Karlijn S. F. M. Hermans, Inez Myin-Germeys, Charlotte Gayer-Anderson, Matthew J. Kempton, Lucia Valmaggia, Philip McGuire, Robin M. Murray, Philippa Garety, Til Wykes, Craig Morgan, Zuzana Kasanova, Ulrich Reininghaus

**Affiliations:** 1 Department of Neuroscience, Center for Contextual Psychiatry, Catholic University of Leuven, Leuven, Belgium; 2 Health Service and Population Research Department, Centre for Epidemiology and Public Health, Institute of Psychiatry, Psychology & Neuroscience, King's College London, London, UK; 3 Department of Psychosis Studies, Institute of Psychiatry, Psychology & Neuroscience, King's College London, London, UK; 4 Psychology Department, Institute of Psychiatry, Psychology and Neuroscience, King's College London, London, UK; 5 National Institute for Health Research (NIHR) Mental Health Biomedical Research Centre at South London and Maudsley NHS Foundation Trust and King's College London, London, UK; 6 Department of Public Mental Health, Central Institute of Mental Health, Medical Faculty Mannheim, University of Heidelberg, Heidelberg, Germany

**Keywords:** altered affective experience, (social) anhedonia, asociality, experience sampling method

## Abstract

**Background:**

It remains poorly understood how negative symptoms are experienced in the daily lives of individuals in the early stages of psychosis. We aimed to investigate whether altered affective experience, anhedonia, social anhedonia, and asociality were more pronounced in individuals with an at-risk mental state for psychosis (ARMS) and individuals with first-episode psychosis (FEP) than in controls.

**Methods:**

We used the experience sampling methodology (ESM) to assess negative symptoms, as they occurred in the daily life of 51 individuals with FEP and 46 ARMS, compared with 53 controls.

**Results:**

Multilevel linear regression analyses showed no overall evidence for a blunting of affective experience. There was some evidence for anhedonia in FEP but not in ARMS, as shown by a smaller increase of positive affect (*B*_Δat−risk *v.* FEP_ = 0.08, *p* = 0.006) as the pleasantness of activities increased. Against our expectations, no evidence was found for greater social anhedonia in any group. FEP were more often alone (57%) than ARMS (38%) and controls (35%) but appraisals of the social situation did not point to asociality.

**Conclusions:**

Overall, altered affective experience, anhedonia, social anhedonia and asociality seem to play less of a role in the daily life of individuals in the early stages of psychosis than previously assumed. With the experience of affect and pleasure in daily life being largely intact, changing social situations and appraisals thereof should be further investigated to prevent development or deterioration of negative symptoms.

## Introduction

Negative symptoms have been reported to strongly impact functioning and burden in patients diagnosed with psychotic disorders (Bobes, Arango, Garcia-Garcia, Rejas, & Group, 2010; Kirkpatrick, Fenton, Carpenter, & Marder, 2006). In individuals with the first episode of psychosis (hereafter referred to as FEP), the prevalence of negative symptoms, primarily measured with the Scale for Assessment of Negative Symptoms (Andreasen, 1989), ranges from 50 to 90% (Makinen, Miettunen, Isohanni, & Koponen, 2008), but seems to reflect, at least in part, presence of comorbid depressive disorder and extrapyramidal symptoms (Malla et al., 2002). Negative symptoms are also more prevalent in individuals with an at-risk mental state (ARMS; also known as ultra-high-risk states) for psychosis (hereafter referred to ARMS) (Fusar-Poli et al., 2013; Valmaggia et al., 2013; Velthorst et al., 2009; Yung et al., 2005) than in controls. Studies so far have demonstrated that patients with psychosis have the capacity to self-report about negative symptoms using cross-sectional questionnaires (Bucci & Galderisi, 2017; Engel & Lincoln, 2017), but also that standardised self-report questionnaires and lab measures do not seem to converge with what is reported in real life (Cohen, Najolia, Brown, & Minor, 2011 Kring & Caponigro, 2010;). This underscores the need for research to investigate individuals' subjective experience of negative symptoms in real time, particularly in comparing ARMS to individuals who have developed a first psychotic episode.

Experience sampling methodology (ESM) has been used to measure negative symptoms in daily life in patients with a psychotic disorder, requiring translation of negative symptoms as used by clinicians and observers to self-report of experience (Myin-Germeys et al., 2018)^1^. Previous ESM studies have investigated altered affective experience and drive as operationalisations of blunted affect, anhedonia, and asociality (Kwapil, Brown, Silvia, Myin-Germeys, & Barrantes-Vidal, 2012; Oorschot et al., 2013). Regarding altered affective experience, ESM studies have shown a lower intensity (i.e. mean level) of positive affect and higher intensity of negative affect in participants with enduring psychotic disorder compared with controls. With respect to instability of affect (i.e. differences in affect from one moment to the next), one study found a higher instability of negative affect and no difference in instability of positive affect (Myin-Germeys, Delespaul, & Devries, 2000), whereas another study found the opposite to hold true, i.e. a higher instability of positive affect and no difference in instability of negative affect between patients and controls (Oorschot et al., 2013). Variability (i.e. differences between affect at the moment and the average individual affect) was found to be lower for positive affect and higher for negative affect in patients compared with controls (Myin-Germeys et al., 2000). Recent work on affect dynamic measures showed that variability of affect is a particularly relevant aspect of well-being in addition to the average intensity levels of affect, and that variability and instability are strongly correlated (Dejonckheere et al., 2019). As both variability and instability have been used in previous ESM studies in enduring psychosis (Myin-Germeys et al., 2000; Oorschot et al., 2013), providing mixed results, it would be important to study these again in early psychosis samples. However, to date, no ESM study has investigated altered affective experience including variability and instability in ARMS.

Several ESM studies have investigated anhedonia in daily life, operationalised as a lower increase of positive affect as a function of increasing pleasantness of events or activities (Oorschot et al., 2013). Findings from these studies suggest that, in individuals with psychosis, there is no momentary anhedonia – reflecting the incapacity to experience pleasure at the moment – but anticipatory anhedonia – reflecting the incapacity to experience pleasure in anticipation of future events (Cohen et al., 2011 Edwards, Cella, Tarrier, & Wykes, 2015; Gard, Kring, Gard, Horan, & Green, 2007; Kring & Caponigro, 2010;). Individuals with psychotic disorder with low levels of negative symptoms even showed a higher increase of positive affect in response to pleasant events compared with controls (Oorschot et al., 2013). In ARMS, laboratory assessments have indicated diminished momentary pleasure compared with controls (Jhung et al., 2016; Strauss, Ruiz, Visser, Crespo, & Dickinson, 2018). Strauss et al. (2018) measured the neurophysiological and self-reported response to emotional stimuli and found a lower intensity of both positive and negative affect in this group. It, therefore, remains to be elucidated whether ARMS and FEP deviate in their hedonic capacity from patients with enduring psychosis in daily life.

Similar to the assessment of anhedonia, social anhedonia assessed with ESM has been operationalised as a lower increase of positive affect as a function of (pleasant) company (Oorschot et al., 2013). ESM studies have found high levels of social anhedonia assessed with a self-report questionnaire to be associated with higher positive affect when alone in daily life (Kwapil et al., 2009), and with lower levels of positive affect in daily life in general, independent of being in company or alone (Brown, Silvia, Myin-Germeys, & Kwapil, 2007). Another ESM study showed similar levels of positive affect in social situations in patients with psychosis and controls, but a stronger desire to be alone in patients than controls (Oorschot et al., 2013). In sum, while being with others has not been consistently linked with lower levels of positive affect in patients with a psychotic disorder, this remains to be investigated in ARMS and FEP.

Regarding asociality, defined as a lack of social drive or interest in social interactions (Blanchard, Collins, Aghevli, Leung, & Cohen, 2011), Kwapil et al. (2009) found a preference to be alone when in company and a desire to be alone when alone to be associated with lower social contact scores in college students with high self-reported social anhedonia. Social withdrawal has also been reported to be prevalent in at-risk samples (Addington, Penn, Woods, Addington, & Perkins, 2008; Piskulic et al., 2012), and to contribute to a lower quality of life and a higher probability of developing or maintaining psychotic symptoms (Robustelli, Newberry, Whisman, & Mittal, 2017). We will investigate social isolation and interest in social interactions in both ARMS and FEP.

The study of negative symptoms and, in particular, the role of social experience in daily life in comparing groups in the early stages of psychosis are important for identifying potential targets for treatment. However, findings are limited and mixed. Especially the focus on ARMS has been unaddressed in previous work on social experience in daily life, but very important given its role in the development of symptoms (Valmaggia et al., 2013). The current ESM study, therefore, aimed to investigate whether negative symptoms were more pronounced in ARMS and FEP than in controls. In order to separately assess this for each negative symptom in daily life, we sought to test the following hypotheses: (H1**)** intensity, variability and instability of positive and negative affect are lower in FEP and ARMS than in controls; (H2**)** pleasantness of events/activities is associated with less intense positive affect in FEP and ARMS compared with controls; (H3**)** (a) company of other people or (b) the appraisal of such company as pleasant is associated with less intense positive affect in FEP and ARMS than in controls; and (H4**)** the amount of time being alone and the preference for being alone is greater in FEP and ARMS than in controls.

## Methods

### Participants

Between June 2012 and August 2014, we recruited ARMS and FEP, identified in the Childhood Adversity and Psychosis study (Gayer-Anderson et al., 2020; Morgan et al., in preparation) and the London centre of The European Network of National Networks studying Gene–Environment Interactions in Schizophrenia (EU-GEI, 2014). The FEP sample was recruited from mental health services in South-East London, UK , and the ARMS sample from a clinical service for people at high risk of psychosis (Outreach and Support in South London; OASIS), the West London Mental Health NHS Trust (WLMHT), as well as a community survey of general practitioner (GP) practices. The control sample was selected using GP lists and the national postal address file. Inclusion and exclusion criteria are listed in Table 1.

**Table 1.** Inclusion and exclusion criteria for FEP, ARMS, and controls

FEP	
Inclusion criteria	Aged 18–64
	Resident within defined catchment areas
	First diagnosis of schizophrenia, schizoaffective, delusional, other non-mood psychotic disorder, or major depressive or manic disorder with psychotic features (ICD-10 F20-F29 and F30-F33) (World Health Organization, 1992), based on the OPCRIT (McGuffin et al., 1991; Reininghaus et al., 2016*a*)
	Adequate command of the English language
Exclusion criteria	Transient psychotic symptoms following acute intoxication
	Psychotic symptoms triggered by an organic cause
ARMS	
Inclusion criteria	Aged 18–35
	Presence of an ARMS based on the CAARMS (Yung et al., 2005) or the SPI-A (Schultze-Lutter et al., 2012)
	Adequate command of the English language
Exclusion criteria	IQ < 60, measured with an adapted version of the WAIS (EU-GEI, 2014; Ryan, Weilage, & Spaulding, 1999)^a^
	Previous experience of psychotic episode for more than 1 week, assessed with the CAARMS (Yung et al., 2005)and the SCID (First, Gibbon, & Williams, 2002)
	Prior use of antipsychotic medication to treat a psychotic episode
Controls	
Inclusion criteria	Aged 18–64
	Resident within the same defined catchment areas as the FEP sample
	Adequate command of the English language
Exclusion criteria	Personal or family history of psychotic disorder (Maxwell, 1992)
	Presence of psychotic experiences, measured with the Psychosis Screening Questionnaire (Bebbington & Nayani, 1995)
	Presence of an ARMS based on the CAARMS (Yung et al., 2005) or SPI-A (Schultze-Lutter et al., 2012)

FEP, first-episode psychosis; ARMS, at-risk mental state for psychosis; OPCRIT, OPerational CRITeria system; WAIS, Wechsler Adult Intelligence Scale; CAARMS, comprehensive assessment of at-risk mental state; SPI-A, Schizophrenia Proneness Instrument – Adult; SCID, structured clinical interview for DSM Disorders.

a The IQ exclusion criterion was not used in each group as ESM data collection for the FEP group and controls was conducted as part of the Childhood Adversity and Psychosis study (Gayer-Anderson et al., 2020; Morgan et al., in preparation) and, for the ARMS group, as part of the prodromal work package of EU-GEI (2014). The IQ criterion was used in the latter but not the former.

### Data collection

#### Basic sample characteristics

Sociodemographic data on age, gender, ethnicity, level of education, and employment status were obtained using a modified version of the Medical Research Council (MRC) sociodemographic schedule (Mallet, 1997). Diagnoses in the FEP sample were based on the OPerational CRITeria system (OPCRIT) (McGuffin, Farmer, & Harvey, 1991; Rucker et al., 2011).

#### Experience Sampling Method

Participants were provided with a study device (Psymate^®^) (Myin-Germeys, Birchwood, & Kwapil, 2011) that prompted them with signals (i.e. beeps) to complete brief questionnaires during six consecutive days. The beeps were emitted ten times a day between 7.30 am and 10.30 pm, at random moments within set blocks of time. Participants were excluded from analysis if they responded to fewer than one-third of the emitted beeps (Palmier-Claus et al., 2011). A detailed description of the ESM measures is shown in Table 2.

**Table 2.** ESM Compliance Procedure^a^ and Measures of affect, anhedonia, social anhedonia, and asociality

Domain	Experience sampling measures
Positive affect	Mean of affect variables ‘I feel cheerful’, ‘I feel enthusiastic’, ‘I feel relaxed’, and ‘I feel satisfied’ (1–7 point Likert scale). Cronbach's alpha is 0.81. We used both high and low physiological arousal items, supported by several studies (Dejonckheere et al., 2019 McManus, Siegel, & Nakamura, 2018;).
Negative affect	Mean of affect variables ‘I feel anxious’, ‘I feel down’, ‘I feel lonely’, ‘I feel insecure’, and ‘I feel annoyed’ (1–7 point Likert scale). Cronbach's alpha is 0.86.
Altered affective experience	Variability: Squared difference between beep-level intensity of positive/negative affect at each observation and individual mean positive/negative affect over observations, over days within persons. Instability: Squared difference between beep-level positive and negative affect intensity at beep t and beep-level positive affect intensity at beep t-1 (previous beep), within days, within persons (MSSD) and only calculated if there were maximum two observations missing between two consecutive observations. Difference scores between two observations overnight were excluded.
Anhedonia (events)	At each beep, participants were asked to ‘think about the most important event that happened since the last beep’. Pleasantness of this event was rated on a bipolar scale ranging from -3 ‘very unpleasant’ to 3 ‘very pleasant’. Anhedonia was defined as the relationship between positive affect and the occurrence of pleasant events. As anhedonia is per definition related to pleasant events, only ratings of 1–3 were used to test associations with positive affect. Observations that indicated unpleasant events (−3 to −1) were excluded from the analysis and neutral events (0) were set as the reference category (Oorschot et al., 2013).
Anhedonia (activities)	The association between positive affect and ‘I like this activity’ in the current moment. The activity referred to was the activity selected as a response to the item ‘What are you doing now?’ (1–7 Likert scale). The distinction between ‘events’ and ‘activities’ was meant to probe for separate experiences.
Social anhedonia	The association between positive affect and being in company (0 being alone, 1 being with others) (Oorschot et al., 2013). The association between positive affect and pleasantness of being in company, rated on a 7-point Likert scale. The item used for pleasantness of being in company was ‘I find being with these people pleasant’.
Asociality	Time spent alone in percentage of total time (time spent alone and time spent in company). Appraisals of being alone or in company were rated on a 7-point Likert scale: If alone: ‘I find it pleasant to be alone’ and ‘I would prefer to have company’. If in company: ‘I find being with these people pleasant’ and ‘I would prefer to be alone’.

a *ESM compliance procedure*: Participants were instructed to respond to the ESM questionnaire within 10 minutes after the signal. They were contacted at least once during the assessment period in order to optimise adherence to the protocol and relieve potential distress related to it. In a debriefing session, reactivity to and compliance with the protocol were assessed.

### Statistical analysis

Statistical analyses were performed using STATA 14.2 (StataCorp., 2015). ESM data have a hierarchical structure with multiple observations (level 1) nested within individuals (level 2). Multilevel linear mixed models were applied to take this into account (Bolger & Laurenceau, 2013; Hox, 2002). Baseline characteristics of each group were compared using ANOVAs for continuous outcomes, and χ^2^-square tests for categorical variables. The models for testing each hypothesis were fitted with restricted maximum likelihood (REML) estimation using the mixed command. This produces unbiased estimates, provided data are missing at random and all variables associated with missing values are included in the model. We conducted group comparisons on intensity, variability, and instability of positive and negative affect in order to investigate altered affective experience (H1). We tested separate associations between pleasantness of events, activities (anhedonia), company (*v*. alone), and pleasantness of being in company (social anhedonia) as independent variables and intensity of positive affect as an outcome. For each association, two-way interactions with the group were added to test for group differences between the associations. If *p* for the interaction was less than 0.05, the ‘lincom’ command was used to compute linear combinations of coefficients and test associations in each group (H2 and H3). All analyses were adjusted for fixed effects of person-level variables (adj^a^: age, gender, ethnicity, level of education, and employment status). In order to investigate confounding of associations by mood, analyses relating to H2 and H3 were repeated while adjusting for feeling down (adj^b^: ESM item ‘I feel down’). Lastly, we conducted group comparisons on variables assessing preference to be alone (when in company), preference to have company (when alone), and pleasantness to be alone (when alone) in order to measure asociality. Time spent in company or alone was computed for each group (H4).

## Results

### Basic sample characteristics

A total of 165 participants provided data with the ESM, of whom 59 were individuals with FEP, 51 were ARMS, and 55 were controls. Eight FEP, five ARMS, and two controls were excluded from the analysis because of incomplete or invalid ESM assessments based on a minimum requirement of 20 valid responses. This resulted in 150 participants (90.9%) being included in the analysis, with a slightly higher number of controls providing valid data. A detailed description of the sample and averages of outcome variables are presented in Supplementary Table S1.

Based on the OPCRIT (McGuffin et al., 1991; Rucker et al., 2011), a diagnosis of psychotic disorder comprised schizophrenia, schizoaffective, or delusional disorder (non-affective psychosis) in 43.9% of the cases, manic or depressive psychosis (affective psychosis) in 29.2%, and psychotic disorder not otherwise specified in 27.1% of the cases.

### H1-Altered affective experience

The intensity of positive affect was, on average, lower and intensity of negative affect was higher in ARMS and FEP compared with controls, with no difference between ARMS and FEP. Variability of positive and negative affect was markedly higher in ARMS compared with controls. With respect to the instability of positive and negative affect, both ARMS and FEP showed markedly higher instability than controls, and this did not differ between ARMS and FEP. Differences between groups in intensity and variability of positive affect and intensity of negative affect remained statistically significant after adjusting for person-level variables (adj.^a^) while some group differences on instability and variability of negative affect, and instability of positive affect were no longer significant (*p* > 0.05) after adjusting for these variables (Table 3).

**Table 3.** Group differences in intensity, variability, and instability of positive and negative affect

	Intensity			Variability			Instability		
	B (s.e.)	95% CI	*p*	B (s.e.)	95% CI	*p*	B (s.e.)	95% CI	*p*
Positive affect									
ARMS – controls									
Unadj	−0.69 (0.17)	−1.01 to −0.36	<0.001	0.49 (0.14)	0.23 to 0.76	<0.001	0.63 (0.22)	0.21 to 1.05	0.003
Adj^a^	−0.58 (0.20)	−0.98 to −0.19	0.004	0.35 (0.17)	0.02 to 0.68	0.038	0.28 (0.26)	−0.24 to 0.80	0.289
FEP – controls									
Unadj	−0.47 (0.16)	−0.15 to −0.79	0.004	0.25 (0.13)	−0.01 to 0.51	0.062	0.46 (0.21)	0.05 to 0.88	0.028
Adj^a^	−0.50 (0.19)	−0.88 to −0.12	0.010	0.20 (0.16)	−0.11 to −0.52	0.206	0.30 (0.25)	−0.20 to 0.80	0.240
ARMS – FEP									
Unadj	−0.22 (0.17)	−0.55 to 0.11	0.191	0.25 (0.14)	−0.03 to 0.52	0.075	0.17 (0.22)	−0.26 to 0.60	0.443
Adj^a^	−0.08 (0.18)	−0.43 to 0.27	0.641	0.14 (0.15)	−0.15 to 0.44	0.336	−0.02 (0.24)	−0.48 to 0.44	0.933
Negative affect									
ARMS – controls									
Unadj	1.10 (0.21)	0.70 to 1.50	<0.001	0.69 (0.13)	0.42 to 0.95	<0.001	1.09 (0.24)	0.63 to 1.56	<0.001
Adj^a^	0.94 (0.25)	0.45 to 1.43	<0.001	0.49 (0.16)	0.18 to 0.80	0.002	0.86 (0.29)	0.30 to 1.43	0.003
FEP – controls									
Unadj	1.10 (0.20)	0.71 to 1.49	<0.001	0.35 (0.13)	0.09 to 0.61	0.007	0.67 (0.23)	0.21 to 1.13	0.004
Adj^a^	0.97 (0.24)	0.50 to 1.44	<0.001	0.21 (0.15)	−0.09 to 0.51	0.175	0.43 (0.28)	−0.11 to 0.98	0.120
ARMS – FEP									
Unadj	0.00 (0.21)	−0.41 to 0.41	0.100	0.33 (0.14)	0.07 to 0.60	0.015	0.42 (0.24)	−0.06 to 0.90	0.083
Adj^a^	−0.03 (0.22)	−0.46 to 0.40	0.896	0.28 (0.14)	0.00 to 0.56	0.046	0.43 (0.26)	−0.07 to 0.93	0.094

B, unstandardised point estimate; s.e., standard error; CI, confidence interval; FEP, first-episode psychosis; ARMS, at-risk mental state for psychosis.

2 Adjusted for person-level variables age, gender, ethnicity, level of education, and employment status.

### H2-Anhedonia

Within each group, positive affect strongly increased with increasing pleasantness of *events*. The association between pleasantness of events and positive affect (as an indicator of anhedonia) was similar in FEP, ARMS, and controls (Supplementary Fig. S1). There was no significant interaction effect of group × pleasant events on positive affect.

**Fig. 1.**
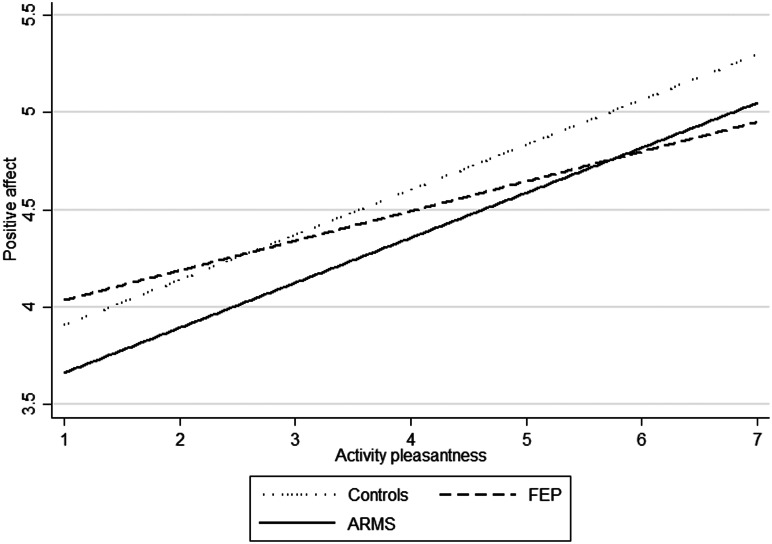
Anhedonia for each Group: positive affect as a function of activity pleasantness.

Within each group, positive affect also increased as the pleasantness of *activities* increased. There was some evidence that the association between pleasantness of activities in the current moment and positive affect did differ across groups (Fig. 1), with the increase in positive affect associated with the pleasantness of activities being smaller in FEP than in controls and smaller in FEP than in ARMS.

### H3-Social anhedonia

Within each group, the intensity of positive affect was higher when in company compared with being alone. As shown in Table 4, there was some evidence that the association between being in company (*v*. alone) and positive affect was different across the three groups, with a larger increase in positive affect when in company compared with being alone in FEP than in controls. There was no evidence for a difference in magnitude of associations when comparing ARMS and FEP nor when comparing ARMS and controls. After adjusting for feeling down, the association between company and positive affect was attenuated in FEP and ARMS, and the interaction of group × company no longer met the conventional cut-off point for ‘statistical significance’ (*p* = 0.05).

**Table 4.** Difference in associations across groups for company, and appraisals of company

	ARMS – controls			FEP – controls			ARMS – FEP		
	B (s.e.)	95% CI	*p*	B (s.e.)	95% CI	*p*	B (s.e.)	95% CI	*p*
Company									
Adj^a^	0.12 (0.07)	−0.02 to 0.25	0.095	0.18 (0.07)	0.05 to 0.32	0.009	−0.07 (0.08)	−0.21 to 0.08	0.382
Adj^b^	0.01 (0.06)	−0.12 to 0.13	0.894	0.10 (0.06)	−0.03 to 0.23	0.123	−0.09 (0.07)	−0.23 to 0.04	0.189
Preference to be alone									
Unadj	0.39 (0.23)	−0.05 to 0.83	0.085	0.55 (0.22)	0.11 to 0.99	0.015	−0.16 (0.23)	−0.62 to 0.30	0.500
Adj^a^	0.45 (0.28)	−0.11 to 1.00	0.112	0.49 (0.27)	−0.05 to 1.02	0.074	−0.04 (0.25)	−0.54 to 0.46	0.875
Preference to have company									
Unadj	0.48 (0.27)	−0.06 to 1.02	0.080	0.85 (0.27)	0.32 to 1.37	0.001	−0.36 (0.28)	−0.91 to 0.18	0.190
Adj^a^	0.22 (0.34)	−0.45 to 0.90	0.519	0.61 (0.33)	−0.03 to 1.25	0.063	−0.39 (0.31)	−0.99 to 0.21	0.203
Pleasantness to be alone									
Unadj	−0.26 (0.24)	−0.72 to 0.21	0.280	−0.90 (0.23)	−1.35 to −0.45	<0.001	0.65 (0.24)	0.17 to 1.12	0.007
Adj^a^	0.101 (0.29)	−0.47 to 0.67	0.727	−0.63 (0.27)	−1.17 to −0.09	0.022	0.73 (0.26)	0.23 to 1.23	0.004

B, unstandardised point estimate; s.e., standard error; CI, confidence interval; FEP, first-episode psychosis; ARMS, at-risk mental state for psychosis; df, degrees of freedom; LR, likelihood ratio.

2 Adjusted for person-level variables age, gender, ethnicity, level of education, and employment status.

3 Adjusted for person-level variables (age, gender, ethnicity, level of education, and employment status) and ESM item ‘I feel down’.

Within each group, more pleasant appraisals of company were associated with a marked increase in the intensity of positive affect. There was no evidence of an interaction effect of group × pleasant company on positive affect, indicating that the association between pleasantness of being in company and positive affect was similar across groups.

### H4-asociality

Controls and at-risk individuals were, on average, 35% and 38% of the time alone, while FEP were, on average, 57% of the time alone. There were group differences regarding preference to be alone when in company, preference to have company when alone, and pleasantness of being alone (Supplementary Table S1). When in company, FEP preferred to be alone more than controls, while, being alone, FEP preferred having company more than controls. Between ARMS and controls and between ARMS and FEP, no statistically significant differences in preference for being alone or preference for having company were found. FEP experienced being alone as less pleasant than controls and as less pleasant than ARMS. Evidence for differences between groups for the pleasantness of being alone held after adjusting for person-level variables (adj.^a^), while findings on preference for being alone and preference for having company were attenuated and fell short of significance (Table 4).

## Discussion

This study showed no overall evidence for a blunting of affective experience, nor for greater social anhedonia in the early stages of psychosis. There was some evidence for anhedonia during current activities in FEP but not in ARMS. Although FEP were more often alone than ARMS and controls, appraisals of the social situation did not point to asociality.

### Methodological considerations

First, ESM data on positive affect, and pleasantness of events, activities, and company were modelled cross-sectionally. Therefore, we cannot exclude the possibility that an increase of positive affect impacted being in company or appraisals of current activities and social situations. Possibilities in advanced mobile technology (e.g. mobility assessment and physiological measures) may help to pinpoint the order of events in real time (Moran, Culbreth, & Barch, 2017).

Second, adjustments for depression were not optimal. Particularly in the at-risk phase, negative symptoms might reflect more general vulnerability for affective symptoms (Strauss et al., 2018). Although we adjusted the analyses controlling for the ESM item ‘I feel down’, we cannot be sure that this fully captured the vulnerability for affective symptoms. This item did attenuate some findings, indicating some overlap between negative and depressive symptoms, especially in ARMS. Further, we did not control for potential comorbid social anxiety disorder, which would be worthwhile to study in the light of the ambiguous appraisals regarding social situations and the social behaviour we found in FEP, especially given the plausible effect anxiety has in a group facing psychotic experiences for the first time.

Third, we formulated distinct hypotheses for different domains of negative symptoms given previous research reported mixed findings on these symptoms. Hence, while acknowledging potential overlap between particularly anhedonia and social anhedonia, we would argue that multiple testing reflects not an issue. Moreover, even if Bonferroni correction (*α* = 0.0125) would have been applied here, the intensity of positive affect in the FEP group was still higher when in company than when being alone. This group's difference with the other groups, however, did not hold and would require further scrutiny.

Fourth, although a slightly higher number of controls provided valid data compared with the other groups, it is unlikely that negative symptoms prevented participants from complying with the ESM procedure as this included regular checks via phone. Another ESM study also showed the number of valid ESM reports to be similar in controls and patients with enduring psychosis and high levels of negative symptoms (Oorschot et al., 2013).

A last potential limitation is using only the at-risk individuals who were selected based on the presence of positive symptoms using the CAARMS (Yung et al., 2005) or the SPI-A (Schultze-Lutter et al., 2012) for the ARMS sample. The additional use of an instrument to assess negative symptoms would have allowed for selecting at-risk individuals with a defined minimum level of negative symptoms. However, this would have limited generalisability of findings to the entire population of at-risk individuals and would not have allowed for investigating the full range of fluctuations in the intensity of negative symptoms in daily life.

### Altered affective experience

We found no evidence for altered affective *experience*, which is in line with other ESM findings, showing higher levels of negative and lower levels of positive affect in patients compared with controls (Cho et al., 2017; Oorschot et al., 2013), and therefore extended this to ARMS and FEP. These findings seem to contrast previous studies reporting a prevalence of blunted affective *expression* in up to 21% of ARMS (Azar et al., 2018 Lepage, Sauvé, Shah, & Brodeur, 2017;), and reports of blunted affective *expression* being a core persisting symptom in patients with (first episode) psychosis (Galderisi et al., 2013; Kirkpatrick et al., 2006). Indeed, recent studies suggested that observer-rated blunted affect and the subjective experience of emotional range reflect two distinct conceptual aspects of negative symptoms (Bucci & Galderisi, 2017; Jang et al., 2016), implying these are not two sides of the same coin. With regard to affective *experience,* we also found markedly higher variability and instability of affect in ARMS, which echoes previous ESM findings by Oorschot et al. (2013), who found lower levels of negative symptoms to be associated with higher instability in patients with non-affective psychotic disorder. The higher variability of positive and negative affect may also result from increased emotional reactivity to different socio-environmental contexts in daily life, which has been repeatedly demonstrated in the literature on stress reactivity using ESM (Myin-Germeys & Van Os, 2007; Reininghaus et al., 2016*b*; Van Der Steen et al., 2017). Given the differences in blunted expression and affective experience, early intervention should target both expression and experience, and increase awareness of potential discrepancies, for instance by enquiring about the experience of affect if expression seems blunted. The use of antipsychotics might have impacted affective blunting and social withdrawal in FEP as all but one were not antipsychotic-naïve. One might speculate that some of the differences across groups may have been due to differences in the use of antipsychotics. Taken together, studies triangulating various measurement modalities (e.g. ESM, experimental tasks, observer ratings) to assess different aspects of affective experience (e.g. affective blunting, emotional reactivity) are now urgently needed to more fully understand its nature in early psychosis.

### Anhedonia

The clinical focus on anhedonia as evidenced by its prominent role in negative symptom scales has been challenged by laboratory and experience sampling research, which point to an intact capacity to experience positive affect from current pleasurable stimuli (Gard et al., 2007; Strauss, Frost, Lee, & Gold, 2017; Strauss, Wilbur, Warren, August, & Gold, 2011). In line with this, we found no evidence of consummatory anhedonia (i.e. lack of positive affect experienced in or just after the moment of the pleasurable activity) with events reported since the last signal. However, our findings were suggestive of some consummatory anhedonia associated with current activities in FEP. This might be interpreted in light of a deficit in ‘positivity offset’ found in patients (Strauss et al., 2017). This experimental finding involved neutral or low-level affective input to occur with lower levels of positive affect in patients compared with controls, who tended to be more positive in neutral or low-level affective activities. Indeed, the average current activity rating of 4.32 in our FEP sample reflects neutral to low affective activities and was not significantly different from the average current activity rating in ARMS and controls.

Our findings in ARMS were in line with other ESM studies (Gard et al., 2007; Oorschot et al., 2013), but at odds with experimental findings showing a hedonic deficit in an at-risk sample (Strauss et al., 2018). Strauss et al. (2018) suggested that this deficit might be explained by comorbidity with symptoms of depression and anxiety (Addington et al., 2011; Fusar-Poli, Nelson, Valmaggia, Yung, & Mcguire, 2014), especially given that the majority will not develop a psychotic disorder (Schultze-Lutter et al., 2015). Although our ESM findings on anhedonia did not support this in ARMS, our findings of higher variability of affect in this group indeed showed similarities to ESM findings in individuals with depression (Heininga, Van Roekel, Ahles, Oldehinkel, & Mezulis, 2017). We controlled for low mood with each beep, but the potential overlap with depression should still be noted and considered in future research. Overall, this suggests that, moving away from the concept of anhedonia as an incapacity to experience positive affect from pleasant stimuli, further exploration of positive affect associated with activities with different levels of affective input may contribute to a deeper understanding of potentially altered social experience in early psychosis.

### Social anhedonia

Similar to anhedonia, social anhedonia is considered a core symptom of a psychotic disorder (Horan, Brown, & Blanchard, 2007; Horan, Green, Kring, & Nuechterlein, 2006). In contrast to findings of social anhedonia in non-clinical samples (Collins, Blanchard, & Biondo, 2005; Kwapil, 1998), at-risk samples (Velthorst et al., 2012) and patients, our findings do not support the presence of social anhedonia in the daily lives of ARMS and FEP. Results even indicate that FEP individuals experienced higher positive affect when being in company than controls, which has also been found in studies using ESM in individuals with persisting subclinical psychotic symptoms (Collip et al., 2014). Other ESM studies investigating social anhedonia in daily life found similar levels of positive affect when being in company in controls and in participants with low and high levels of negative symptoms (Kasanova, Oorschot, & Myin-Germeys, 2018; Oorschot et al., 2013). Discrepancies between ESM and non-ESM findings may be explained by the latter being based on self-reports and clinical interview measures that retrospectively assess positive affect towards social situations and relationships in general (Martin, Cicero, Bailey, Karcher, & Kerns, 2016), whereas ESM measures positive affect in real-life social situations (Kring & Barch, 2014). Overall, ESM studies seem to converge on the notion that people in the early stages are capable of enjoying the presence of others. The next question is how this, then, relates to behaviour.

### Asociality

FEP spent less time in company than ARMS and controls, in concordance with another ESM study (Oorschot et al., 2013). This could potentially be due to the differences in lifestyle among the groups, with FEP having significantly lower employment rates than ARMS who did not differ from controls. That is, FEP likely had less opportunities to be in social company that one tends to engage in as part of a job. A recent study found evidence for asociality in chronic patients only during activities such as work, while no such deficit was observed in more casual social contexts (Kasanova et al., 2018). This change in daily life activities may become particularly pertinent when the first episode of psychosis develops, often leading people to retract from school or their job. In addition to behaviour, our FEP sample showed ambiguity in the appraisals of social situations (i.e. preferring company when alone and vice versa). Following the distinction between contexts made by Kasanova et al. (2018), this may reflect ambiguity towards social situations that individuals could freely choose, and explain the contrast with findings from other ESM studies (Brown et al., 2007; Kwapil et al., 2009; Kwapil et al., 2012), which included college students who typically engage in many structured activities.

Given that the first occurrence of full-blown psychotic symptoms has an important impact on social interactions (Gayer-Anderson & Morgan, 2013), and social anhedonia seems to be absent in our sample, our results may reflect experiences of stigma and change of social roles as described in patients with psychosis (Rusch et al., 2014), which become apparent in individuals at risk for psychosis as well (Yang et al., 2015). This external threat may explain ambiguous appraisals of FEP towards social situations, which would be worthwhile to study in more depth as the onset triggers of deficient and intact emotional experience are still largely unidentified (e.g. Kring & Elis, 2013). The potential of altered experience in the early stages as a response to external threat and its social impact should therefore be addressed in studies with a longitudinal character, investigating the potentially changing impact of social threat in the development of psychosis. In order to prevent internalisation of stigma and social withdrawal, it may be promising to enhance social support as well as help patients to satisfactorily adapt, remain in, or take on again, social roles that can often not be fulfilled anymore (Ramsay et al., 2011). Investigation of a direct link between experiences of stigma and social situations in daily life may be an important next step in order to further our understanding of the impact of emerging symptoms on social interaction in early psychosis.

## Conclusions

Our findings suggest that the experience of pleasure and affect in daily life is intact in the early stages of psychosis. In addition, we found no evidence of social anhedonia when measured in real life. However, more time spent alone in FEP compared with ARMS implies an important difference in their social environment and a potential mismatch between what individuals need (i.e. being around others) and what is actually happening in their real-life social environment (i.e. sustaining social isolation as it is assumed based on individuals' expression). Treatment should, therefore, target individuals and their social environment in order to improve and facilitate the social interaction that they need.

## Data Availability

The original data cannot be openly shared because of security reasons given the sensitivity on data relating to an at-risk mental state for, or a first episode of, psychotic disorder. Since data from this study were in part collected as part of a NIHR Postdoctoral Research Fellowship and an EU FP7 grant, availability to share data also depends on the policy of these funding bodies. Further details on how to apply for the data are available from the corresponding author on request.

## Note

1 ESM is a structured diary technique in which participants keep a dedicated device over several days. At random times throughout the day, this device prompts them to complete brief questionnaires about their current mood, symptoms, context, and appraisals of their context (e.g. Palmier-Claus et al., 2011; Myin-Germeys et al., 2009).

## Supplementary material

For supplementary material accompanying this paper visit https://doi.org/10.1017/S0033291720001154. click here to view supplementary material
