# Supplementary material for: Elucidating negative symptoms in the daily life of individuals in the early stages of psychosis
Source: Psychol Med. 2020 May 22;51(15):2599–609. doi: 10.1017/S0033291720001154 (PMC8579154; doi:10.1017/S0033291720001154)
Supplement: Supplementary file 1 [file S0033291720001154sup.zip › S0033291720001154sup001.docx]

**Supplementary Table S1.** Sociodemographic Characteristics and Group Differences; Average ESM Scores of Outcome Variables per Group, Standard Error; Test for Differences between Groups

|  | **Controls** | **ARMS** | **FEP** | **Test statistic** | **p** |
| --- | --- | --- | --- | --- | --- |
|  | (n=53) | (n=46) | (n=51) |  |  |
| Age in years, mean (SD) | 35.0 (12.6) | 23.6 (4.7) | 28.3 (8.6) | F=18.6, df=2 | <0.001 |
| Gender, n (%) |  |  |  | χ2=1.0, df=2 | 0.612 |
| Men | 25 (47.2) | 21 (45.7) | 28 (54.9) |  |  |
| Women | 28 (52.8) | 25 (54.4) | 23 (45.1) |  |  |
| Ethnicity, n (%) |  |  |  | χ2=14.0, df=10 | 0.174 |
| White British | 25 (47.2) | 17 (37.0) | 14 (27.5) |  |  |
| Black African | 8 (15.1) | 7 (15.2) | 17 (33.3) |  |  |
| Black Caribbean | 6 (11.3) | 7 (15.2) | 11 (21.6) |  |  |
| Asian | 3 (5.7) | 1 (2.2) | 1 (2.0) |  |  |
| White Other | 5 (9.4) | 5 (10.9) | 4 (7.8) |  |  |
| Other | 6 (11.3) | 9 (19.6) | 4 (7.8) |  |  |
| Level of education, n (%) |  |  |  | χ2=24.3, df=4 | <0.001 |
| Primary | 8 (15.1) | 13 (28.9) | 17 (33.3) |  |  |
| Secondary | 15 (28.3) | 24 (53.3) | 25 (49.0) |  |  |
| Higher education | 30 (56.6) | 8 (17.8) | 9 (17.7) |  |  |
| Employment status, n (%) |  |  |  | χ2=28.5, df=2 | <0.001 |
| Unemployed | 5 (9.4) | 15 (32.6) | 30 (58.8) |  |  |
| Employed | 48 (90.6) | 31 (67.4) | 21 (41.2) |  |  |
| Valid beeps per person, mean (SE) | 46.04 (1.10) | 39.91 (1.50) | 37.41 (1.41) | F=11.54, df=2 | <0.001 |
| Intensity positive affect, mean (SE) | 4.46 (0.11) | 3.77 (0.28) | 3.99 (0.27) | χ2=18.36, df=2 | <0.001 |
| Variability positive affect, mean (SE) | 0.73 (0.09) | 1.22 (0.23) | 0.97 (0.23) | χ2=13.20, df=2 | 0.001 |
| Instability positive affect, mean (SE) | 1.08 (0.15) | 1.71 (0.36) | 1.54 (0.36) | χ2=9.49, df=2 | 0.009 |
| Intensity negative affect, mean (SE) | 1.93 (0.14) | 3.02 (0.35) | 3.02 (0.34) | χ2=39.83, df=2 | <0.001 |
| Variability negative affect, mean (SE) | 0.48 (0.09) | 1.17 (0.23) | 0.84 (0.22) | χ2=26.10, df=2 | <0.001 |
| Instability negative affect, mean (SE) | 0.58 (0.16) | 1.67 (0.4) | 1.25 (0.4) | χ2=21.58, df=2 | <0.001 |
| Pleasantness of events, mean (SE) | 1.18 (0.09) | 1.09 (0.23) | 0.88 (0.23) | χ2=5.03, df=2 | 0.081 |
| Pleasantness of activities, mean (SE) | 4.62 (0.12) | 4.35 (0.28) | 4.34 (0.28) | χ2=3.80, df=2 | 0.150 |
| Being alone in %, mean (SE)^1^ | 35.09 (4.13) | 37.77 (4.61) | 57.17 (4.59) | χ2=13.86, df=2 | 0.001 |
| Pleasantness of company, mean (SE) | 5.34 (0.14) | 4.98 (0.34) | 4.87 (0.34) | χ2=6.20, df=2 | 0.045 |
| Pleasantness of being alone, mean (SE) | 4.62 (0.16) | 4.36 (0.4) | 3.71 (0.39) | χ2=16.13, df=2 | <0.001 |
| Preference to be alone, mean (SE) | 2.11 (0.15) | 2.5 (0.38) | 2.66 (0.38) | χ2=6.40, df=2 | 0.041 |
| Preference to have company, mean (SE) | 2.81 (0.19) | 3.3 (0.46) | 3.66 (0.45) | χ2=10.18, df=2 | 0.006 |

*Note:* ARMS, At-Risk Mental State for psychosis; FEP, First-Episode Psychosis; SD, standard deviation; df, degrees of freedom

*Note:* Pleasantness of events ranges from 0 (neutral) to 3 (very pleasant); pleasantness of activities, pleasantness of company, pleasantness of being alone, preference to be alone, and preference to have company range from 1 (not at all) to 7 (very much); being alone in percentage of time

^1^ Binary variable (alone vs. in company) used as an outcome variable in a multilevel logistic regression. Tested for differences between groups in percentage of time spent alone with Bonferroni correction for multiple comparisons.
