# Supplementary material for: Elucidating negative symptoms in the daily life of individuals in the early stages of psychosis
Source: Psychol Med. 2020 May 22;51(15):2599–609. doi: 10.1017/S0033291720001154 (PMC8579154; doi:10.1017/S0033291720001154)

**Supplementary Figure S1.** Anhedonia for each Group: Positive Affect as a Function of Event Pleasantness


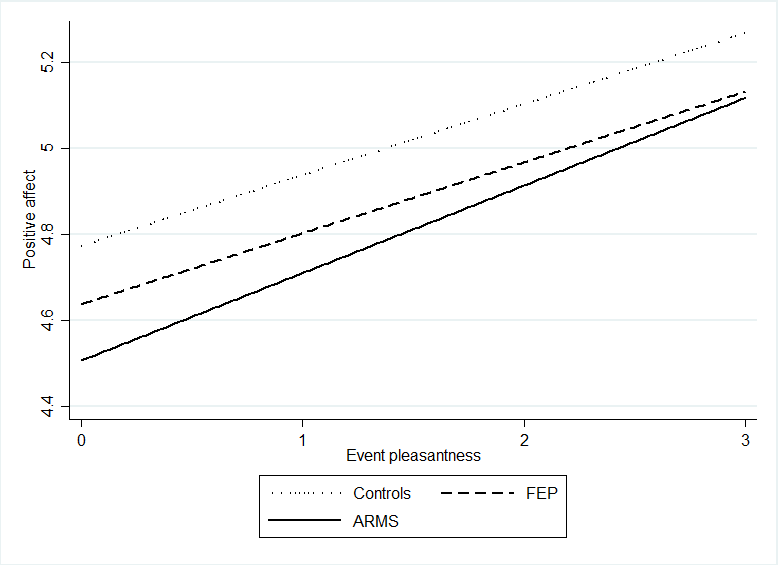

Supplement: Supplementary file 1 [file S0033291720001154sup.zip › S0033291720001154sup002.docx]
